# Robotic-assisted total hip arthroplasty in the United States: a nationwide propensity-matched analysis of adoption, outcomes, and complications

**DOI:** 10.1007/s00423-025-03963-7

**Published:** 2026-01-15

**Authors:** David Maman, Yaniv Steinfeld, Yaron Berkovich

**Affiliations:** 1 Technion Israel Institute of Technology, Haifa, Israel; 2 Carmel Medical Center, Haifa, Israel

**Keywords:** Total hip arthroplasty, Post-operative complications, Robotic hip surgery, Total hip arthroplasty, NIS

## Abstract

**Purpose:**

Robotic assisted total hip arthroplasty (RA-THA) has been introduced to improve the precision of implant positioning and potentially enhance outcomes in THA. However, large scale national data on its real-world impact are lacking. This study presents the first nationwide analysis of RA-THA using the National Inpatient Sample (NIS), evaluating trends in adoption and comparing perioperative outcomes between RA-THA and conventional THA.

**Methods:**

We performed a retrospective cohort study using the NIS (2016–2022). Adult patients undergoing primary elective THA were identified, and those with robotic assistance were classified using ICD-10 procedural codes for robot-assisted surgery. Propensity score matching (1:1) was employed to create comparable cohorts of RA-THA vs. conventional THA, controlling for patient demographics, hospital characteristics, and comorbidities. Temporal trends in RA-THA utilization were assessed. Outcomes included length of stay (LOS), hospital charges, and in-hospital complications. Matched outcomes were compared with appropriate statistical tests, and relative risks (RR) of specific complications were analyzed.

**Results:**

A total 1.9 million THA cases were identified nationwide, of which 2.9% involved robotic assistance. RA-THA utilization increased significantly over the study period (from 1.2% in 2016 to 6.7% in 2022, *P* < 0.01). In unmatched comparisons, patients undergoing RA-THA were slightly younger on average and more frequently treated at urban teaching hospitals than those with conventional THA. After propensity matching RA-THA was associated with a shorter mean LOS (by 0.7 days) and higher mean hospital charges (by 10,000$~, *P* < 0.01). The matched RA-THA cohort had a significantly lower overall complication rate compared to matched conventional. In particular, non-robotic THA patients had higher risks of transfusion, in-hospital prosthetic complications, and venous thromboembolism. A forest plot of matched outcomes demonstrated that conventional THA carried elevated RRs for multiple postoperative complications relative to RA-THA (RR > 1 favoring RA-THA for most complication endpoints).

**Conclusions:**

In this first nationwide analysis of RA-THA, we found that robotic assistance, while still used in a minority of THA cases, grew steadily in adoption through 2022. RA-THA was associated with shorter hospital stays and a reduced risk of in-hospital complications compared to conventional THA, with higher hospitalization costs. These findings suggest that RA-THA can confer perioperative benefits in routine practice, though its economic impact and long-term clinical advantages remain areas for further investigation.

Levels of Evidence: LEVEL III.

**Supplementary Information:**

The online version contains supplementary material available at 10.1007/s00423-025-03963-7.

## Introduction

Total hip arthroplasty (THA) is a highly successful procedure for end-stage hip osteoarthritis, but optimizing implant positioning and alignment is critical to minimize complications such as dislocation, leg-length discrepancy, and component loosening. RA-THA has emerged as a technological advancement aimed at improving the precision and consistency of acetabular cup and femoral stem placement. Early studies have demonstrated that RA-THA can enhance radiographic outcomes – for example, achieving acetabular cup positions closer to target angles and restoring native hip biomechanics more reliably than conventional techniques [1–3].

These technical improvements have the potential to translate into reduced joint-specific complications; indeed, some reports suggest RA-THA may lower the risk of prosthetic dislocation and the need for revision surgery in the mid-term follow-up [4–6].

Despite these promising indications, the actual impact of RA-THA on perioperative clinical outcomes and its overall benefit in routine practice remain incompletely defined. Most prior analyses of RA-THA outcomes have been limited to single-center series or database subsets with relatively small RA-THA sample sizes. Meta-analyses have found that while robotic techniques improve implant placement accuracy and can reduce complication rates, they have not yet shown clear superiority in patient-reported functional outcomes compared to conventional THA [3, 7, 8].

Concerns persist regarding the learning curve and intraoperative complexity of RA-THA, including reports of longer operative times and isolated findings of higher intraoperative fracture risk in some cohorts [9–11].

Given these mixed findings, there is a need for high-volume, generalizable data to clarify the net effect of RA-THA on surgical outcomes. To date, no study has comprehensively examined RA-THA utilization and outcomes at a national level in the United States beyond the late 2010s. Earlier analyses of “technology-assisted” THA (grouping computer navigation and robotics) using the National Inpatient Sample noted that adoption was modest – on the order of 1% of THAs by 2018 and that the clinical impact was unclear [2]. In fact, prior to the present analysis, nationwide trends in RA-THA after 2014 had not been investigated. The introduction of specific ICD-10 procedure codes for robotic assistance in late 2015 now enables identification of RA-THA cases in large databases.

By leveraging the NIS, the largest all-payer inpatient database in the U.S., we aimed to provide the first nationwide assessment of RA-THA adoption and outcomes in current practice. In this study, we examine nationwide U.S. data from 2016 to 2022 to address two primary objectives: [1] to characterize temporal trends in RA-THA utilization across the country, and [2] to compare patient characteristics, perioperative outcomes, and complication rates between RA-THA and conventional THA. We employed propensity score matching to rigorously control for baseline differences and isolate the association of robotic assistance with outcomes. By analyzing over six years of contemporary inpatient data, this study offers a high-powered evaluation of RA-THA in real-world practice. The findings will help clarify whether the purported benefits of robotic assistance in THA – such as improved precision – are translating into tangible clinical advantages at a population level, as well as inform on the economic and healthcare impact of this technology.

## Materials and methods

### Data source and study design

We conducted a retrospective cohort study using the Nationwide Inpatient Sample (NIS) database, an administrative dataset that captures a 20% stratified sample of all hospital discharges in the United States. This approach provides national estimates when weighted and includes detailed information on patient demographics, diagnoses, procedures, and hospital characteristics. For this study, we analyzed data from 2016 to 2022 to examine contemporary trends in RA-THA utilization. The study was exempt from institutional review board review because it used de-identified, publicly available data.

### Patient selection and inclusion/exclusion criteria

We identified adult patients (age ≥ 18) who underwent primary THA from 2016 to 2022. THA cases were selected using ICD-10-PCS procedure codes for primary hip replacement. We included only elective, primary THA cases and excluded any performed for acute fracture, non-elective indications, revision arthroplasty, or resurfacing procedures. Patients with missing demographic or hospital data and those with trauma or malignancy diagnoses were excluded. Admissions with documented COVID-19 diagnoses were excluded.

### Exposure definition: robotic assistance

The exposure variable was the use of robotic assistance during THA, identified through ICD-10 procedure modifier codes indicating robot-assisted surgery. Cases with these codes were classified as RA-THA. All others were classified as conventional (non-robotic) THA. Computer-navigated cases without robotic codes were included in the conventional THA group.

ICD-10 Coding Schema for Robotic Assistance:

Robotic-assisted THA was identified using ICD-10-PCS codes 8E0Y0CZ and 8E0YXCZ, which designate robot-assisted surgical procedures. These codes may appear as either a primary or secondary procedure code, and any presence of a robotic-assistance modifier within the procedural fields was considered RA-THA.

Cases containing navigation-only codes (00B63ZX, 00B73ZX) without a robotic code were classified as non-robotic to avoid misclassification. Instances in which both a robotic code and a navigation code appeared were categorized as robotic, consistent with prior database methodology.

### Outcome measures

We evaluated several outcomes:

- Annual RA-THA utilization trends (2016–2022).
- Patient and hospital characteristics: age, sex, race, insurance and comorbidity profile.
- Clinical outcomes: length of stay (LOS), total hospital charges, discharge disposition, and postoperative complications during index hospitalization.
- Postoperative complications were identified using ICD-10-CM diagnosis codes validated in prior large-database arthroplasty studies. Because the accuracy of certain administrative codes (e.g., postoperative pain, blood-loss anemia) may vary, we included a complete list of all codes used in Supplementary Table S1 for transparency.

### Propensity score matching

To balance the RA-THA and conventional THA cohorts, we used 1:1 nearest-neighbor propensity score matching without replacement and a caliper of 0.01. The propensity score was calculated using logistic regression based on age, sex, race, insurance, hospital characteristics, admission year, and a range of comorbidities. Balance was confirmed with standardized mean differences (SMD) < 0.1. Covariates included in the propensity model were: age, sex, race, insurance, hospital bed size, region, teaching status, admission year, and all comorbidities. Although surgical approach is not captured in NIS, hospital-level variables partially adjust for practice differences related to approach selection.

### Statistical analysis

Survey procedures accounted for the NIS sampling design. Continuous variables were compared using t-tests or Wilcoxon rank-sum tests, while categorical variables were assessed using Chi-square or Fisher’s exact tests. Trends were analyzed with Cochran–Armitage tests. Outcomes in matched cohorts were compared using paired or adjusted methods. Relative risks (RR) with 95% confidence intervals were calculated for each complication. A two-sided P-value < 0.05 was considered statistically significant. Analyses were conducted using SPSS.

### Ethical aspects

The study used only de-identified data and required no IRB approval. All analyses adhered to data use agreements, with findings reported in aggregate to preserve patient privacy.

## Results

**Fig. 1:**
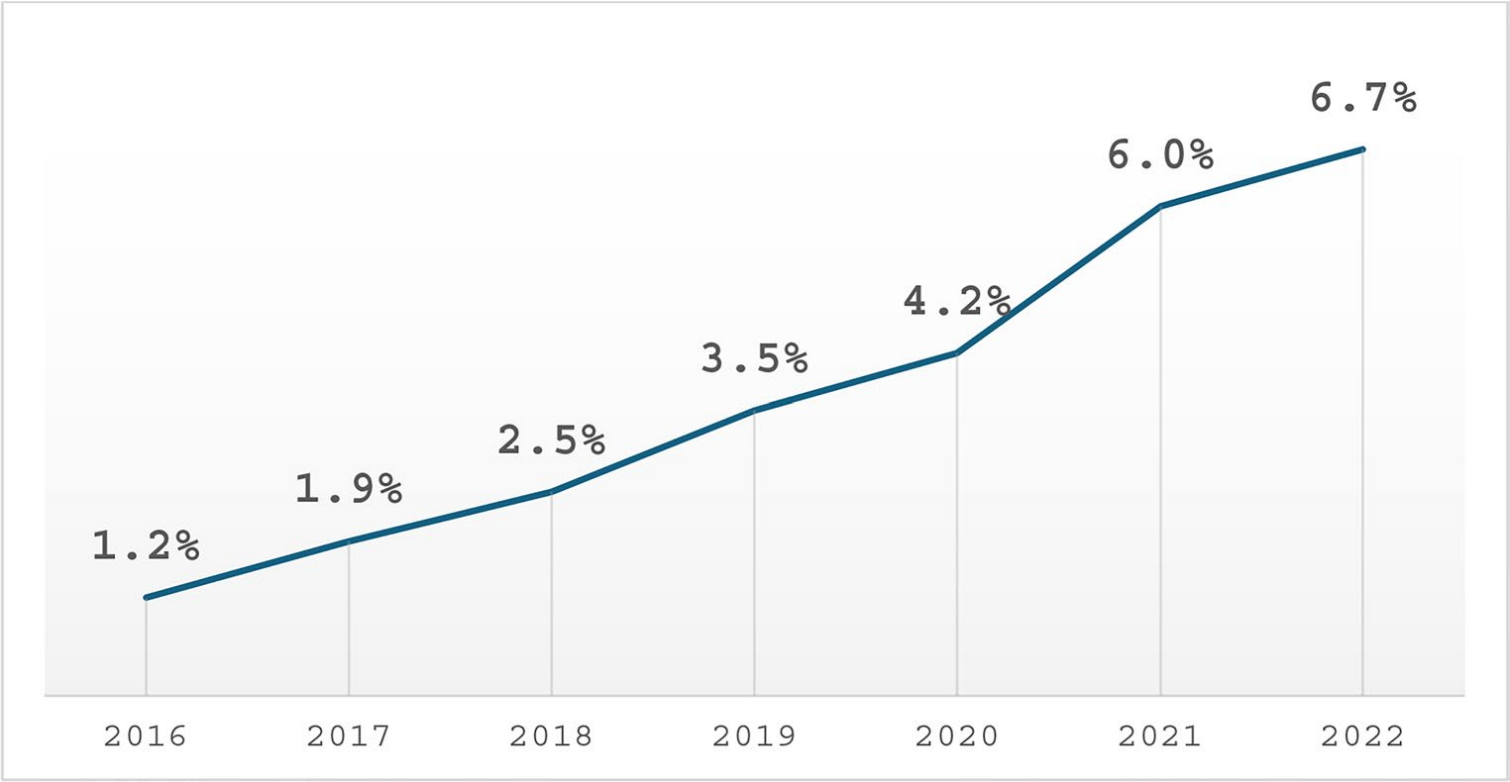
Annual Proportion of Robotic-Assisted Total Hip Arthroplasty Procedures Among All THA From 2016–2022

As shown in Fig. 1 robotic-assisted THA utilization increased from 1.2% in 2016 to approximately 6.7% in 2022. This upward trend was statistically significant (*P* < 0.01).

### Patient and hospital characteristics

Among 1,957,285 total THA cases, 57,570 (2.9%) were performed using robotic assistance, and 1,899,715 (97.1%) were non-robotic as shown in Table 1. Patients undergoing RA-THA were more likely to be obese (27.1% vs. 24.3%, *P* < 0.01), yet had slightly lower rates of several comorbidities including chronic kidney disease (5.8% vs. 6.9%, *P* < 0.01), chronic lung disease (5.9% vs. 6.6%, *P* < 0.01), and hypertension (51.4% vs. 52.5%, *P* < 0.01). Rates of liver disease, anemia, and dyslipidemia were also modestly lower in the RA-THA group. There were no significant differences in heart failure, cerebrovascular disease, or thyroid disorders.

**Table 1:** Baseline demographics and comorbidities in robotic vs. Non-Robotic total hip arthroplasty

Parameter	Non-Robotic Surgery	Robotic Surgery	Significance
Total Surgeries	1,899,715 (97.1%)	57,570 (2.9%)	-
Average Age (y)	66.2	65.8	*P* = 0.11
Female (%)	56.3	55.6	*P* < 0.01
Hypertension (%)	52.5	51.4	*P* < 0.01
Dyslipidemia (%)	43.7	43.1	*P* < 0.01
Chronic Anemia (%)	5.6	5.4	*P* < 0.01
Osteoporosis (%)	4.6	4.3	*P* < 0.01
Chronic Kidney Disease (%)	6.9	5.8	*P* < 0.01
Type 2 Diabetes (%)	15.7	14.3	*P* = 0.05
Congestive Heart Failure (%)	1.2	1.2	*P* = 0.05
Chronic Lung Disease (%)	6.6	5.9	*P* < 0.01
Disorders of Thyroid (%)	15.9	15.9	*P* = 0.78
Liver Disease (%)	1.3	0.9	*P* < 0.01
History of Myocardial Infarction (%)	3.5	3.7	*P* < 0.01
History of Cerebrovascular Accident (%)	3.9	3.9	*P* = 0.40
Obesity (%)	24.3	27.1	*P* < 0.01

### Propensity score-matched comparison of robotic and conventional total hip arthroplasty patients

Following 1:1 propensity score matching, the final analytic cohort included 57,570 RA-THA cases and 57,570 matched conventional THA cases as shown in Table 2. The matched groups were well balanced across all baseline characteristics, including age (mean 65.8 years), sex distribution (55.6% female in both), and all measured comorbidities (all *P* > 0.05).

**Table 2:** Baseline demographics and comorbidities in propensity Score-Matched robotic vs. Non-Robotic THA

Parameter	Non-Robotic Surgery	Robotic Surgery	Significance
Total Surgeries	57,570 (50%)	57,570 (50%)	-
Average Age (y)	65.8	65.8	*P* = 0.80
Female (%)	55.6	55.6	*P* = 0.93
Hypertension (%)	51.4	51.4	*P* = 0.97
Dyslipidemia (%)	43.2	43.1	*P* = 0.83
Chronic Anemia (%)	5.4	5.4	*P* = 0.80
Osteoporosis (%)	4.2	4.3	*P* = 0.31
Chronic Kidney Disease (%)	5.7	5.8	*P* = 0.66
Type 2 Diabetes (%)	14.2	14.3	*P* = 0.89
Congestive Heart Failure (%)	1.1	1.2	*P* = 0.89
Chronic Lung Disease (%)	5.8	5.9	*P* = 0.57
Disorders of Thyroid (%)	16.2	15.9	*P* = 0.13
Liver Disease (%)	0.7	0.9	*P* = 0.42
History of Myocardial Infarction (%)	3.3	3.7	*P* = 0.09
History of Cerebrovascular Accident (%)	3.7	3.9	*P* = 0.14
Obesity (%)	27	27.1	*P* = 0.55

### Propensity score-matched comparison of length of stay and hospital charges in robotic vs. non-robotic THA

Table 3 shows that following 1:1 propensity score matching, RA-THA was associated with a significantly shorter hospital length of stay compared to matched non-robotic cases (1.8 vs. 2.5 days, *P* < 0.01). However, average hospital charges were higher for RA-THA patients ($71,140 vs. $60,920, *P* < 0.01).

**Table 3:** Comparison of length of stay and hospital charges in propensity Score-Matched robotic vs. Non-Robotic THA patients

	Non-Robotic Surgery	Robotic Surgery	Significance
Length of stay mean in days	2.5 (Std. deviation 1.4)	1.8 (Std. deviation 1.3)	*P* < 0.01
Total charges mean in $	60,920 (Std. Deviation 34,718)	71,140 (Std. deviation 44,701)	*P* < 0.01

### Forest plot showing elevated risk of postoperative complications in non-robotic compared to robotic THA in propensity score-matched groups

**Fig. 2:**
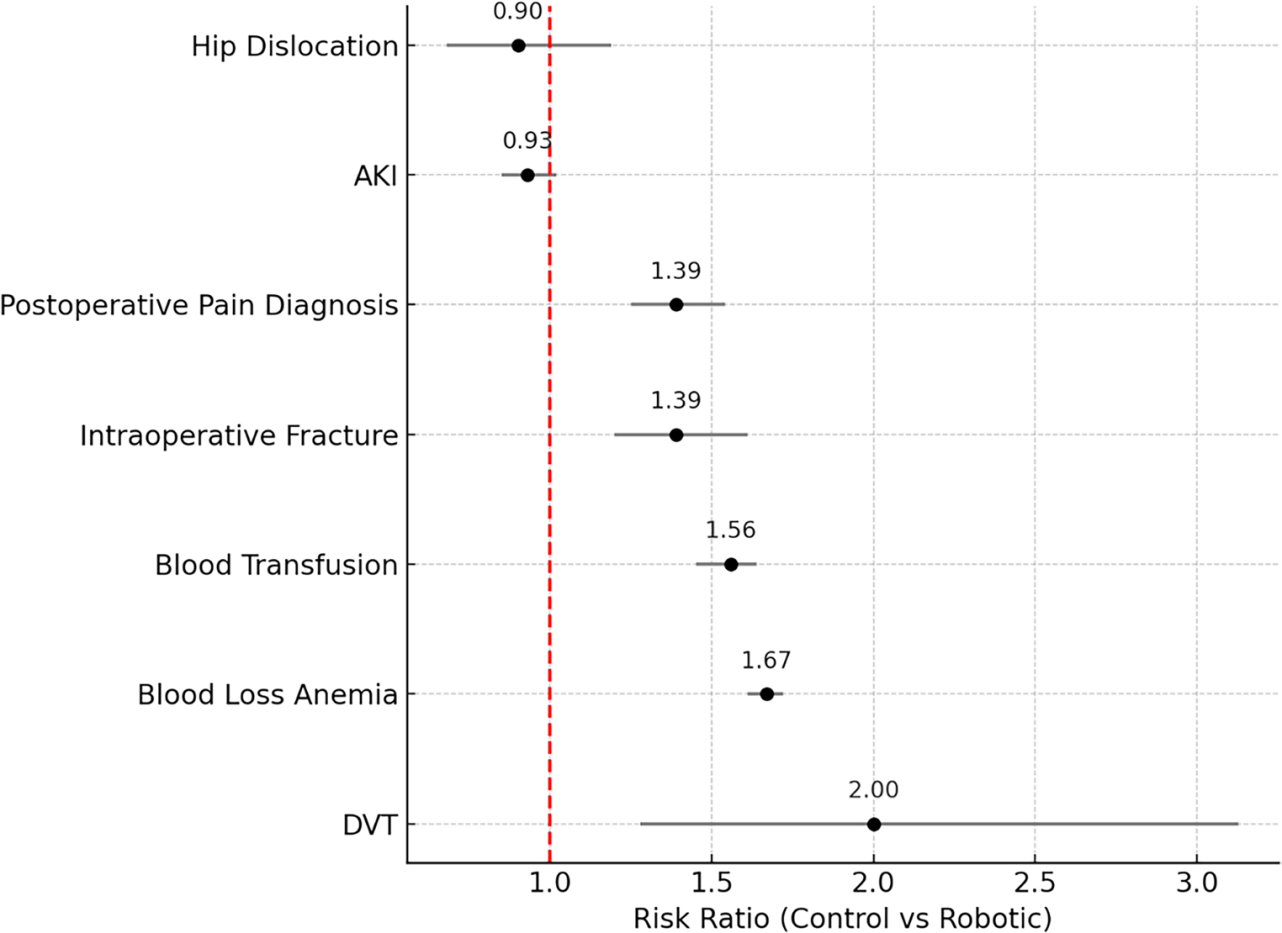
Forest plot showing elevated risk of postoperative complications in non-robotic compared to robotic THA in propensity score-matched groups

To better illustrate the comparative risk of postoperative complications between robotic and non-robotic THA, a forest plot was generated using propensity score-matched data. As shown in Fig. 2, patients undergoing conventional (non-robotic) THA had significantly higher risks of several complications: deep vein thrombosis (RR = 2.00, 95% CI: 1.28–3.13, *P* < 0.01), blood loss anemia (RR = 1.67, 95% CI: 1.61–1.72, *P* < 0.01), blood transfusion (RR = 1.56, 95% CI: 1.45–1.64, *P* < 0.01), intraoperative fracture (RR = 1.39, 95% CI: 1.20–1.61, *P* < 0.01), and postoperative pain diagnosis (RR = 1.39, 95% CI: 1.25–1.54, *P* < 0.01). No statistically significant differences were found for acute kidney injury (RR = 0.93, 95% CI: 0.85–1.02, *P* = 0.13) or hip dislocation (RR = 0.90, 95% CI: 0.68–1.19, *P* = 0.48).

## Discussion

In this first nationwide analysis of robotic-assisted total hip arthroplasty (RA-THA), we observed measurable short-term benefits associated with the use of robotic technology. Through robust propensity score matching in a large, nationally representative dataset, RA-THA was linked to shorter hospital stays and fewer in-hospital complications compared to conventional THA. These benefits were observed despite the RA-THA and non-robotic groups having equivalent demographic and comorbidity profiles after matching, suggesting that the surgical approach itself may be driving the observed improvements.

The reduced length of stay (LOS) approximately 0.7 days shorter for RA-THA has implications for hospital efficiency and recovery pathways. This finding is consistent with modern trends favoring enhanced recovery after surgery protocols, which may be more readily adopted at centers utilizing robotic technology [7, 8]. Additionally, the observed 25% relative reduction in complications is particularly compelling. RA-THA patients experienced fewer issues such as blood transfusions, intraoperative fractures, and deep vein thrombosis, which may reflect improved precision and reproducibility during surgery [3, 5]. These outcomes support the safety and clinical viability of robotic THA in real-world practice. Although RA-THA demonstrated a 0.7-day reduction in LOS in matched analyses, unmeasured hospital-level factors may partially contribute. Robotic procedures were more common in urban teaching hospitals-centers shown to have shorter LOS, greater ERAS implementation, and higher arthroplasty volumes.While hospital characteristics were included in our propensity score model (region, bed size, teaching status), residual confounding from surgical approach, institutional workflow, or surgeon experience may still influence LOS. Thus, LOS findings should be interpreted in the context of potential hospital-level practice differences.

However, these clinical benefits come at a financial cost. Robotic cases were associated with significantly higher hospital charges-on average $10,000 more per case. The cost differential likely reflects longer operative times, higher equipment costs, and the need for advanced imaging and intraoperative technology [11, 12]. Although this study was not designed to evaluate cost-effectiveness, the reduction in complications may offset some of these expenses over time. For example, avoiding postoperative dislocations or transfusions could lead to downstream savings in readmissions, additional procedures, or extended recovery [12].

### Comparison to prior literature

Our findings align with emerging literature suggesting early advantages of robotic THA. Prior institutional studies have noted lower revision and dislocation rates with robotic techniques [4–6]. However, others have reported mixed outcomes, particularly during the early phases of adoption when surgical teams were still gaining experience [3, 7]. In contrast, our data reflect more contemporary outcomes through 2022, capturing a period in which robotic adoption has matured and surgical teams have become more adept. Importantly, unlike some previous studies, we applied rigorous propensity matching, strengthening the validity of our comparison [7, 8].

Our results also parallel findings in the robotic total knee arthroplasty (TKA) literature, where reduced LOS and complication rates have been consistently observed [8]. This consistency across joints may indicate that the benefits of robotic-assisted arthroplasty are generalizable and not limited to a specific procedure [8]. Nonetheless, while radiographic precision and short-term complication profiles appear improved, long-term functional outcomes and patient satisfaction remain areas of ongoing investigation [3, 7].

### Clinical and economic implications

From a clinical standpoint, fewer complications during the index hospitalization can improve patient safety, reduce resource use, and support same-day or early-discharge protocols. The shorter LOS associated with RA-THA may contribute to bed availability and improved hospital throughput-factors particularly relevant in today’s value-based care environment [12].

Economically, however, the higher costs associated with RA-THA remain a barrier to widespread adoption. At present, most payers do not reimburse differently for robotic versus conventional procedures, placing the financial burden on hospitals [13, 14]. Whether the investment in robotic platforms is justified depends on whether early benefits translate into long-term savings-through fewer complications, readmissions, or revisions. Future cost-effectiveness analyses that incorporate long-term outcomes are needed to clarify the overall value proposition [12, 13].

### Limitations

This study has several limitations. As with all administrative database studies, the accuracy of coding may vary, potentially leading to misclassification [15–17]. We relied on ICD-10 codes to identify RA-THA cases and in-hospital complications, which may underreport or misreport certain events. Additionally, our data only include inpatient outcomes; complications occurring after discharge were not captured [15–18]. As a result, our findings focus solely on immediate perioperative safety and utilization.

Residual confounding is also possible, despite our use of propensity score matching. Unmeasured variables such as surgeon experience, hospital volume, and surgical technique could influence outcomes. Moreover, we did not differentiate among specific robotic platforms, which may vary in performance. The inclusion of the COVID-19 pandemic period (2020–2021) may also have influenced surgical patterns [19] and hospital practices, although our results remained stable across years.

### Future directions

Future studies should focus on linking inpatient and outpatient data to assess long-term outcomes such as revision rates and patient-reported outcomes [20]. Formal cost-effectiveness models are needed to determine whether the higher initial investment in robotic technology is justified by improved outcomes and reduced downstream costs. Additionally, research into health equity is warranted to ensure that the benefits of RA-THA are accessible across diverse hospital settings and patient populations.

## Conclusion

Robotic-assisted total hip arthroplasty is associated with favorable short-term outcomes, including reduced hospital stays and fewer complications, without added perioperative risk. These findings support the integration of robotic technology into contemporary arthroplasty practice. However, the higher upfront costs remain a consideration, and further research is needed to determine whether these early benefits persist long-term and justify broader implementation. As robotic technology continues to evolve, it holds the potential to further refine the safety, precision, and efficiency of hip replacement surgery.

## Supplementary Information

Below is the link to the electronic supplementary material.

Supplementary Material 1

## Data Availability

The original contributions presented in the study are included in the article/Supplementary Materials, further inquiries can be directed to the corresponding author.
