# Supplementary material for: Robotic-assisted total hip arthroplasty in the United States: a nationwide propensity-matched analysis of adoption, outcomes, and complications
Source: Langenbecks Arch Surg. 2026 Jan 15;411(1):65. doi: 10.1007/s00423-025-03963-7 (PMC12835064; doi:10.1007/s00423-025-03963-7)
Supplement: Supplementary file 1 — Supplementary Material 1 [file 423_2025_3963_MOESM1_ESM.docx]

| **Condition** | **ICD-10-CM Codes** |
| --- | --- |
| **Heart Failure / Congestive Heart Failure** | I5021, I5031, I5033, I5041, I5043, I500, I501, I509 |
| **Acute Kidney Injury (AKI)** | N170, N171, N172, N178, N179 |
| **Acute Coronary Syndrome / Myocardial Infarction** | I2101, I2102, I2109, I211, I2111, I2119, I212, I2129, I213, I214, I219 |
| **Stroke / Cerebrovascular Disease** | I60, I61, I62, I63, I650, I688, O873, O2250, O2251, O2252 |
| **Pulmonary Edema** | J810, J811, I501 |
| **Hypertension** | I10* (all codes beginning with I10) |
| **Blood Loss Anemia** | D62* (all codes beginning with D62) |
| **Chronic Anemia** | D64* (all codes beginning with D64) |
| **Dyslipidemia** | E78* (all codes beginning with E78) |
| **Pneumonia** | J189, J159, J22 |
| **Venous Thromboembolism (Pulmonary Embolism)** | I2602, I2609, I2692, I2699 |
| **Deep Vein Thrombosis (DVT)** | I82401, I82402, I82403, I82409, I82411, I82412, I82413, I82419, I82421, I82422, I82423, I82429 |
| **Obstructive Sleep Apnea** | G473 |
| **Osteoporosis** | M81, M82 |
| **Alcohol Use Disorder** | F10 |
| **Mental Disorders (general category)** | F* (all codes beginning with F) |
| **Parkinson Disease** | G20* |
| **Type 2 Diabetes Mellitus** | E11* |
| **Chronic Kidney Disease (CKD)** | N18* |
| **Chronic Lung Disease (COPD, etc.)** | J44* |
